# Correlates of Active Commuting to School among Portuguese Adolescents: An Ecological Model Approach

**DOI:** 10.3390/ijerph19052733

**Published:** 2022-02-26

**Authors:** Nuno Loureiro, Vânia Loureiro, Alberto Grao-Cruces, João Martins, Margarida Gaspar de Matos

**Affiliations:** 1 Projeto Aventura Social, Faculdade de Motricidade Humana, Universidade de Lisboa, Estrada da Costa, 1499-002 Cruz Quebrada, Portugal; nloureiro@ipbeja.pt (N.L.); mmatos@fmh.ulisboa.pt (M.G.d.M.); 2 ISAMB/Faculdade de Medicina, Centro de Investigação Apoiado Pela Universidade de Lisboa, 1649-028 Lisboa, Portugal; jmartins@fmh.ulisboa.pt; 3 Departamento de Artes, Humanidades e Desporto, Instituto Politécnico de Beja, 7800-295 Beja, Portugal; 4 GALENO Research Group, Department of Physical Education, Faculty of Education Sciences, University of Cadiz, 11519 Puerto Real, Spain; alberto.grao@uca.es; 5 Instituto de Investigación e Innovación Biomédica de Cádiz (INiBICA), 11009 Cadiz, Spain; 6 Centro de Estudos de Educação, Faculdade de Motricidade Humana e UIDEF, Instituto de Educação, Universidade de Lisboa, 1649-004 Lisboa, Portugal; 7 CIPER, Faculdade de Motricidade Humana, Universidade de Lisboa, Estrada da Costa, 1499-002 Cruz Quebrada, Portugal

**Keywords:** teenagers, active transportation, walking, family, social support, financial level

## Abstract

Active commuting to and from school can be an important contribution to improving health in adolescents. This study aimed to analyze the influence of multilevel variables of the ecological model in the active commuting of a representative sample of Portuguese adolescents. The 2018 Health Behaviour in School-Aged Children questionnaire was applied to 5695 adolescents with an average age of 15.5 years old (SD ± 1.8), 53.9% of whom were girls. The associations were studied by applying chi-square tests and multivariate logistic regression models. In this study, 36.5% of the participants reported walking or cycling to school. Active commuting to school was directly associated with age (OR = 1.2; *p* < 0.05), strong family support (OR = 1.2; *p* < 0.05), a moderate to low financial level of the family (OR = 1.3; *p* < 0.05) and living near the school (OR = 2.4; *p* < 0.05). The results revealed that an adolescent’s choice to travel to and from school using an active mode of transportation increased with strong family support. As a result, promotion campaigns should consider the adolescent’s family context.

## 1. Introduction

Physical activity (PA) is an important behavior for the health of adolescents and for a healthy lifestyle [1]. Several studies have provided compelling evidence that regular PA produces health benefits, such as mental health benefits [2], reduction in cardiovascular risk factors [3], physical fitness and well-being [4], and social health benefits [5]. More recently, a review [6] confirmed the impact of moderate- to vigorous-intensity PA on brain health, especially in the neurophysiological and structural aspects of the human brain, such as improved brain plasticity, particularly through changes in brain-derived neurotrophic factor, functional connectivity, basal ganglia, and the hippocampus. However, globally, it is estimated that only some adolescents (19%) are active enough [7] to achieve the recommendation of 60 min of moderate-to-vigorous PA (MVPA) a day [8]. In Portugal, these values are even lower in boys (14%) [9] and especially in girls aged 15–17 years as only 5% comply with the recommendations [10].

Active commuting (AC), which can be defined as the use of active means such as walking and cycling, is considered an important means for the accumulation of PA and to achieve daily recommendations [11,12,13] and one of the eight investments that work for PA [14]. AC also helps reduce air pollution and carbon emissions while benefitting the national economy [15]. One review [4] identified positive associations between AC and health outcomes across 68 studies. A systematic review conducted by Henriques-Neto, et al. [16] revealed positive effects or relationships between AC and several attributes of physical fitness. In addition to the impact of AC on young people’s individual PA levels and health, the promotion of AC is also seen as a transversal relevant strategy with the potential to contribute toward several sustainable development goals, such as those related to quality education, good health, sustainable cities and communities, and climate action [17]. However, the prevalence of AC is low and seems to be decreasing in the last decades in several countries. For example, it decreased in China from 83.8% in 2002 to 54.3% in 2012 [18] and from 48% in 1969 to 13% in 2009 in the USA [19], with similar results in other developed countries such as Canada [20], Australia [21], Ireland [22], Spain [23], and Portugal [24].

The ecological vision is based on the idea that the individual does not develop in a vacuum and one of the most comprehensive ecological models for active transport [25] includes intrapersonal, household, environmental, behavioral, policy, and social influences. This multidimensional approach integrates the social and physical environment as well as accepting that personal aspects are important elements in health behavior [26]. More recently, Mandic, et al. [27] reported that AC is a complex behavior influenced by multiple factors including personal (sociodemographic, behavioral patterns, motivational factors, and perceived barriers), social (peer support, family resources, and school factors) and environmental (urban/rural setting, distance to school, and neighborhood safety perceptions). As young people age, they gain a greater degree of autonomy and, to some extent, parental concerns are reduced [28]; however, this prevalence decreases with increasing age [29].

The role of parents is crucial to determining adolescents’ mode of transport to school [30]. Parental concerns, such as traffic safety and social support, are relevant factors in the encouragement and permission of AC to school [31]. Low socioeconomic status [32] and fewer vehicles at home [33] are positively correlated with AC. Conversely, the perceptions of parents about longer distance, traffic safety, convenience, built environment, crime-related safety, and weather are related to lower AC among children [34]. The results of the Aranda-Balboa, Chillón, Saucedo-Araujo, Molina-García and Huertas-Delgado [34] showed the need to modify the perceptions of children and their parents to increase AC.

The commuting distance, an environmental factor, was one of the strongest correlations for AC adoption between adolescents [27]. A longer route to school may be perceived as less safe than the route to a local park [28] and have less adult supervision [35].

The main aim of this study was to understand the influence of multilevel variables of the ecological model in the active commuting of Portuguese adolescents.

## 2. Materials and Methods

### 2.1. Participants and Procedures

We used data from research conducted in Portugal in 2018 within the scope of the Health Behaviour in School-Aged Children (HBSC) in collaboration with the World Health Organization (WHO) Regional Office for Europe [36]. The HBSC is a cross-sectional study that takes place in 44 countries and regions across Europe and North America and surveys adolescents’ health and health behaviors. The main goal is to enhance the understanding of youth health and well-being, their health behaviors, and their social contexts. Based on a questionnaire with the same questions to all participant countries, the study is carried out every four years, making it possible to compare the indicators between countries and to understand their evolution within each country.

The participants were enrolled via a clustered sampling design (the sampling unit was the class) to meet significant sample of 8th- (13–14 years old) and 12th-grade students (>17 years old) who attend regular education in continental Portugal according to standard guidelines from the HBSC/WHO survey protocol [37]. The schools provided their authorization, legal guardians provided signed informed consent, and students gave assent.

The questionnaires were answered anonymously, voluntarily, and completed in an online format during class time by trained teachers. Filling out the questionnaire took about 60 min. This investigation had the approval of the Ethical Committee of Porto Medical School, the National Data Protection System, and the Portuguese Ministry of Education.

Sampling was conducted using IBM SPSS Statistics 25 software (IBM, Armonk, NY, USA) with a PPS design (probability proportional to size), which took into account the school size and distribution (in percentage) of the students for the included grades in the corresponding regions, stratified by type of school [38]. As a result, a representative sample of the Portuguese school population, 42 school groups from all over the continental country (5 schools per region) and a total of 387 classes, was selected. This study involved 5695 Portuguese students (53.9% girls, M_age_ = 15.5 ± 1.8 years): 8th grade (*n* = 2766, M_age_ = 14 ± 0.83 years), 10th grade (*n* = 1711, M_age_ = 16.1 ± 0.9 years), and 12th grade (*n* = 1218, M_age_ = 18.0 ± 0.8 years).

### 2.2. Measures

#### 2.2.1. Active Commuting to School

To determine the AC, we focused on the answers of the questionnaire, which investigated how the adolescents travelled to school. The HBSC questions used were the following: “How do you go from home to school every day (and from school to home)?” with the following options: (a) public transportation; (b) car; (c) motorcycle; (d) walking; (e) bicycle; (f) other. The responses were divided dichotomously: those who answered “walking” or “bicycle” were classified as students who regularly engaged in AC to school, whereas those who answered “public transportation”, “car”, or “motorcycle” were classified as individuals who did not engage in AC to school. The students who answered “other” were excluded to minimize possible misclassification. This question is one of the most appropriate to ask about the mode of commuting to school [39] and is considered a valid and reliable measure [40].

#### 2.2.2. Individual Correlations

Participants provided information on their age (date of birth), gender (male/female), and schooling level (8th, 10th, and 12th grades).

#### 2.2.3. Social Correlates

The perception of family support was obtained by the question “My family really tries to help me”. Participants selected the answer that shows the degree of agreement or disagreement from “very strongly disagree” (1) to “very strongly agree” (7). The results were recoded into binary variables (0–4 as 0 (weak) to moderate vs. 5–7 as 1 (strong)). This question usually integrates the multidimensional scale of perceived social support, which presents good validity and reliability [41].

The support of friends was obtained through the question “The following figure represents a ladder. The top of the stairs is ‘10’ and represents a very good relationship with your friends, the bottom of the stairs is ‘0’ and represents a very bad relationship with your friends. Right now, where do you think you are on the stairs?”. The answers were recoded as 0 for a good relationship and 1 for a very good relationship.

To know the perception of the family’s financial level, we used the following question: “How do you think your family is financially?” The possible answers ranged from very good (1) to very bad (5) and were recoded using dichotomous variables 0 (bad to average (3–5)) and 1 (good (1–2)).

#### 2.2.4. Environmental Correlations

The proximity of the student’s home to school was evaluated with the question “How much time do you take from your home to your school? Consider the house where you live most of the time”. According to the McDonald, et al. [42] recommendation, the variable was dichotomized in “near” (less or equal to 16 min) or “far away” (longer than 16 min).

### 2.3. Statistical Analyses

Descriptive analysis of all variables was undertaken for the total sample. The chi-square test (χ^2^) was used to determine the statistically significant differences between AC and the individual characteristics (sex, age, schooling, and mode of commuting), social support (family, friends, and financial level of the family) and environmental variables (school proximity). To determine relevant differences between variables, the value ≥ |1.9| of adjusted residual was considered. Binary multilevel logistic regression analyses were carried out to assess the relationships between AC and individual (sex, age, schooling, and mode of commuting), social support (family, relationship with friends, and financial level of the family), and environmental (proximity of the school) variables. The results are presented as numbers, proportions, and/or odds ratios (OR) and their 95% confidence intervals (95% CI). A *p*-value of <0.05 was considered statistically significant. All statistical analyses were carried out in Statistical Package for Social Sciences, version 27.0 (IBM SPSS Inc., Chicago, IL, USA).

## 3. Results

The descriptive characteristics of the study sample are presented in Table 1. We verified that 53.9% of the participants were girls and 34.9% were 16 years old or older. We found that 36.5% reported choosing AC when travelling to school. A total of 64% of adolescents had strong family support and 60.2% had a good relationship with friends. An estimated 54.9% of adolescents declared that their family had a low to moderate economic level, and 73.7% lived near the school.

Table 2 shows the results of the chi-square test between mode of commuting and individual characteristics (sex, age, and schooling), family support, relationship with friends, financial level of the family, and school proximity. Significant differences were identified among the 10th- to 12th-grade students (38.7%) and older adolescents (41.6%) had higher percentages of using an AC mode for traveling to school. Overall, 39.3% of students with moderate family support, a low to average financial level of the family (39.7%), and who lived near the school (41.1%) were more likely to use AC. No significant differences were identified in gender and the relationship with friends.

Table 3 shows the adjusted OR values of the statistical multivariate logistic regression, showing the variables associated with AC. Older adolescents (OR = 1.2; *p* < 0.05) and those from 10th to 12th grade (OR = 2; *p* < 0.05) were associated with higher odds of performing more AC. The adolescents who reported strong support from their families also showed stronger associations (OR = 1.2; *p* < 0.05) of using AC modes for traveling to school. The perception of a low financial level of the family (OR = 1.3; *p* < 0.05) and living near school (OR = 2.4; *p* < 0.05) also contributed to increasing the probability of AC. No associations were found between the relationship with friends and AC.

## 4. Discussion

The main aim of this study was to understand the influence of multilevel variables on the ecological model in the active commuting (AC) of Portuguese adolescents. The results suggest that AC tends to be higher in older adolescents, adolescents with stronger family support, a lower financial level of the family, and in those who lived near the school.

The results show a 36.5% prevalence of AC to school. These results are similar to the 37.5% of Brazilian adolescents [43] but lower than the 54.3% of the Chinese adolescents [18]. At the national level, these results are in line with those of previous Portuguese HBSC studies (e.g., 35% in Loureiro et al. [44]). Thus, AC among Portuguese youth seems to be stable across the years and needs to be improved.

The central point of ecological models is that all levels of influence matter, so it is important to discuss these results to understand the influence of different multilevel factors in adoption of AC by adolescents. On the intrapersonal level, we could not find an association between sex and AC. These results are different from those found by Gong, Yuan, Feng, Ma, Zhang, Ding, Chen and Liu [18], where girls were significantly more likely to perform AC. However, these results are not unanimous [22,45] and should be interpreted with caution. Nevertheless, AC can be an important strategy to increase girls’ physical activity levels, particularly in countries with low levels of AC such as Portugal [36]. Regarding age, we identified that AC has a higher probability to be performed more often by older adolescents. Age is an important moderator for adolescent AC since older adolescents may have fewer personal safety concerns due to greater autonomy and less dependence on parents [46], especially in the case of girls [43]. Furthermore, they have less parental control, which significantly contributes to the adolescents’ decisions about AC [47]. Older age can also result in possible access to private motorized transport, which might impact AC, especially in families with lower socioeconomic status [23].

In our study, the adolescents who reported strong support from their families showed stronger associations with attending school in an active way. One of the most important barriers to AC for teenagers was motivational and social support barriers [34].

Our findings suggest that adolescents need physical or motivational encouragement and support to walk or cycle to school. It is essential to develop interventions linked to specific contexts, as barriers for parents of children and parents of adolescents are similar but not the same and, consequently, interventions in school and high school may differ [34]. Subsequently, we also highlight the need to design detailed interventions that involve both populations (parents and adolescents children) [34]. At this level, it is also important to consider that, in this study, AC involved two behaviors: walking and cycling. Previous studies [44,48] showed that the prevalence of Portuguese adolescents cycling to school is very low (ranging from 1% to 5%). This indicates that there is a need and an opportunity for a specific intervention to promote AC modes related to cycling. As such, novel approaches are needed. As demonstrated by Chillón, et al. [49], at the school level, we suggest that the curricular physical education and school sport contexts might be relevant contexts for teaching adolescents to learn how to cycle while motivating those adolescents who may have a bicycle but do not use it often in their daily life and for travelling to school.

The literature suggests that adolescents report higher PA levels when they have social support from their parents or/and friends [50]. We found that the perception of a lower financial level of the family increased the adoption of AC. These results are similar to those of another study [18], which showed that participants with higher family incomes and education reported a lower prevalence of AC. This is mainly due the fact that the paradox of traffic [51] and an increase in the purchasing power of families foment the purchase of motorized vehicles, which are used to transport young people in their daily routines. The importance of the economic question can also be extrapolated to the national dimension. The investigation by González, et al. [52] demonstrated that when comparing the prevalence of AC among children and adolescents from 49 countries at different levels of development, countries with a very high Human Development Index have low prevalence of AC and low inequalities, but there are countries, such as the Netherlands, that manage to invert this relation [53]. Thus, people are more likely to travel by private car and motorcycle by reason of convenience, timeliness, and reliability [18].

Our data show that living less than 16 min from school is a strong indicator of the probability of adopting AC. Furthermore, cross-sectional studies have consistently shown distance as a main barrier to AC [34] and it is the strongest variable influencing AC to school among young people [54,55].

This study has some characteristics that we consider pertinent. First, the representativeness of the sample is valuable and the outcomes can be extrapolated to the national context.

Much of the previous literature has been limited to understanding the constraints of AC in countries such as Portugal. In our study, we considered multiple levels consistent with a conceptual framework for adolescent decision making about transport choices [27]. One of the limitations of this study is related to the fact that the questions used did not address the characterization of AC (e.g., times per week and combined modes of transportation), which may have led to interpretation problems for the participants. Family support and relationships with friends are not specific to AC. Another limitation is not including questions related to safe paths to school. Future studies can consider the specific support of parents for AC and study the importance of safe paths to school. Another restraint involved the variables related to perceptions, which are always conditioned by a high degree of subjectivity, for which the complementary use of instruments that assess AC directly is recommended.

## 5. Conclusions

Our results showed that in an ecological model, it is important to understand the variables associated with AC reported by Portuguese adolescents in the 2018 HBSC study. The results showed a 36.5% prevalence of AC. the findings also suggested that the choice of adopting AC modes to school is higher among older adolescents, adolescents with a strong family support, lower financial level, and living near the school. AC may be an important strategy to increase physical activity levels, especially in girls. Its promotion should focus especially on young people who live closer to the school and with the support of parents to attenuate the perceived barriers to the use of AC. Increasing AC to school should be promoted in all levels, not leaving the task only for the parents and children, but also promoting it in local politics (e.g., slow down in the school area signs and lower speed limits) and to the adolescents and their families (e.g., awareness of the importance of AC, not only because of fitness and health benefits but also due to environmental sustainability issues). We think that it is important to contribute to increasing adherence to active transport through crafting a societal message that it is fun, healthy, manageable, and accessible to all. The design or identification of safe paths to school can increase the number of users and consequently create an environment for greater confidence within families.

## Figures and Tables

**Table 1:** Descriptive data of participants and variables used in the investigation (*n* = 5695), HBSC study Portugal, 2018.

Items Studied	*N* *	%
Individual		
Boy	2628	46.1
Girl	3067	53.9
≤13 years	1752	30.8
14–15 years	1948	34.2
≥16 years	1985	34.9
8th grade	2766	48.6
10th grade	1711	30.0
12th grade	1218	21.4
Passive Transportation	2716	63.5
Active commuting	1560	36.5
Social Support		
Weak to moderate family support	1946	36
Strong family support	3457	64
Good relationship with friends	2683	60.2
Very good relationship with friends	1777	39.8
Bad to average financial level of the family	2816	54.9
Good financial level of the family	2316	45.1
Environmental		
Live near (≤16 min) to school	3203	73.7
Live away (>17 min) from school	1143	26.3

* Number completing the survey item.

**Table 2:** Chi-square inferential analysis of the Portuguese adolescents of active commuting (*n* = 5695), HBSC study Portugal, 2018.

Variables	Active Commuting			
	No		Yes	
	*n*	%	*n*	%
Sex				
Female	1534	64.8	833	35.2
Male	1180	61.9	727	38.1
Age *				
13–14 years	844	**69**	380	**31**
15–16 years	914	64.8	497	35.2
>16 years	958	**58.4**	682	**41.6**
Grade *				
8th grade	1251	**66.3**	635	**33.7**
10th to 12th grade	1465	**61.3**	925	**38.7**
Family support *				
Weak to moderate	944	**60.7**	612	**39.3**
Strong	1772	**65.1**	948	**34.9**
Relationship with friends				
Bad to good	1624	63.9	916	36.1
Very good	1050	22.6	628	37.4
Financial level of the family *				
Low to average	1443	**60.3**	949	**39.7**
Good	1273	**67.6**	611	**32.4**
Proximity to school *				
Live Near (≤16 min) to school	1845	**58.9**	1289	**41.1**
Live away (>17 min) from school	837	**76**	264	**24**

* Chi-square significant values for *p* < 0.05; Adjusted residuals ≥|1.9| are considered significant (in bold).

**Table 3:** Explanatory logistic regression of the Portuguese adolescents’ uses of active transport to school (*n* = 1560), HBSC study Portugal, 2018.

Variable	Active Commuting OR (95% CI)
Sex (A)	
Male	0.9 (0.8–1.1)
Age (B)	**1.2 (1.1–1.3)**
Grade (C) *	
≥Tenth grade	**1.4 (1.1–1.7)**
Family support (D)	
Strong	**1.2 (1.1–1.4)**
Financial level of the family (E)	
Bad to average	**1.3 (1.1–1.5)**
Proximity to school (F)	
Live near (<16 min) to school (G)	**2.4 (2.1–2.9)**
Constant	74.328

Note: (A) Girls are the reference group. (B) Age was introduced as a continuous variable. (C) Eighth grade is the reference group. (D) Weak to moderate family support is the reference group. (E) Good financial level of the family is the reference group. (F) “Not always place of residence” is the reference group. (G) living ≥17 min proximity to school is the reference group. Values in bold mean significant results *p* < 0.05; CI indicates confidence intervals; OR means odds ratio.

## Data Availability

The data presented in this study are available at http://aventurasocial.com/ (accessed on 10 March 2021).
